# Annotations

**Published:** 1910-09-17

**Authors:** 


### ANNOTATIONS.

### "Phossy-Jaw."

Americans are in the habit of boasting, and this with a certain amount of reason, of the spirit of progress which animates their manufacturing methods. Yet it is the United States which is practically the only large commercial country that has made no legislative effort to prevent the use of the deadly white phosphorus in the manufacture of matches. It appears that few safety-matches are made in the United States, and that these are produced by a single company possessing patent rights. Most of the matches in common use are made of white phosphorus which, as might be expected, exacts its toll of victims from among workers in the match industry. J. B. Andrews, of the American Association for Labour Legislation found forty cases of "phossy-jaw" recorded as occurring in one of the best factories. In three others eighty-two cases were reported. It is obvious, therefore, that legislation is urgently necessary to cope with this evil. The extra cost which would fall upon the manufacturers by the use of a form of phosphorus free from the poisonous qualities possessed by the white variety, would not, it is computed, exceed 5 per cent. In view of the cheapness of matches in this country where the use of the safe variety of phosphorus is insisted upon by law, it could not be deemed a great hardship to the public of the United States if the increased cost of manufacture were reflected on the consumer by a slight rise in the retail price of matches. It would seem that the company possessing the patent rights of manufacturing safety matches has offered to place them at the disposal of the other companies, providing that the United States Government prohibits the use of white phosphorus in the industry. While this concession is no doubt made largely on commercial grounds, justice to the workers demands that it be accepted, and that the cost of manufacture should be thrown upon consumers and not upon the health of the workpeople. As the *American Journal of Surgery* says, "it is regrettable that this objectionable loss of workers cannot be stopped at once by a country that is so willing to listen to cries against vivisection."

### Preparation 606.

A note of warning, or at least of circumspection, was sounded in these pages very recently (The Hospital, August 20, 1910, p. 602) on the subject of the new organic arsenic compound with which Dr. Paul Ehrlich claims that syphilis can be instantaneously and certainly cured. It is claimed that two thousand patients have thus been treated, and that after one injection it is no longer possible to find the treponema pallidum, though before it has been abundantly demonstrated. The too sceptical attitude, as some readers thought it, of our article seems to be on the way already to be justified. Two Prague experimenters report in the *Wien. Klin. Wochenschrift* fourteen patients treated with "606," in three of whom more or less alarming symptoms arose. Vesical paralysis happened in all three, with retention of urine lasting from twelve hours to nine days; obstinate constipation was present in all these patients, and two of the three had severe tenesmus. Albuminuria appeared twice also. Ehrlich himself has failed to produce any such effects; but in the *Münchener Medizinische Wochenschrift* Alt describes autopsies on two patients who had received the treatment. The chief point of interest disclosed is that the compound may remain unchanged at the site of injection, and not be absorbed at all. The latest development is the advocacy of this preparation for sleeping sickness and other diseases caused by trypanosomes. Without departing from the strictest scientific reserve, the profession in England will note with great interest the results of treatment by this newly-introduced drug, the methods of whose introduction are most unfortunately reminiscent of the aryl-arsonates. It is to be hoped that investigators will publish their experience of it fully and freely, whether that experience be favourable or unfavourable to the merits of "606."

### The Supply of Diphtheria Antitoxin.

With the praiseworthy object of facilitating the prompt supply of antitoxin in cases of diphtheria the Local Government Board on August 17 announced an order which sanctions the provision by Metropolitan Borough Councils of supplies of this remedy to meet the requirements of urgent cases. The Councils are to consult their respective medical officers of health with respect to the storage and distribution of diphtheria antitoxin; and the Board suggests that at an early date the arrangements shall be considered by the Councils and their medical officers, and that prophylactic as well as curative administration shall be germane to the discussion. It is evidently the intention of the Board to interfere with the private medical practitioner as little as possible, because it is expressly laid down that the latter is the proper person in the ordinary way to decide upon and to administer antitoxin. It is especially for Poor Law medical officers that the provision of diphtheria antitoxin is regarded as desirable by the Board. It is matter for congratulation both to the profession and to the Board that at last justice is to be done, or at least rendered possible, over this matter. Hitherto parish medical officers have only too often had to meet out of their own pockets the cost of antitoxin for cases of diphtheria under their charge. It can be affirmed most positively that in numberless instances the meagre salary of these officials, whose work for the sick poor goes too often as unacknowledged as it is ill-rewarded, has been entirely eaten up by the heavy expense of providing diphtheria antitoxin. In such cases—and they have been very common—the more thoroughly conscientious the medical officer is the more heavily is he penalised financially. The provision of such an absolutely essential yet costly drug as diphtheria antitoxin should in common fairness never have been a function of Poor Law medical officers. As a prophylactic, benefiting the whole neighbouring community, as well as a necessary aid to the actual sufferer, antitoxin should be available promptly and freely.

